# Giant Palmar Lipoma: A Rare Presentation

**Published:** 2017-05

**Authors:** Veena Singh, Vijay Kumar, Arun K Singh

**Affiliations:** 1 Department of Plastic and Reconstructive Surgery, AIIMS PATNA, Phulwarisharif, Patna, Bihar, India;; 2 Department of Plastic and Reconstructive Surgery, King George Medical University, Lucknow, Uttar Pradesh, India

**Keywords:** Giant lipoma, Deep palmar space

**DEAR EDITOR**

Lipomas are common tumors that arise from mesenchymal fibrofatty tissue. They are the single most common soft tissue tumor^1^ and can involve any part of the body. The most common clinical presentation is a gradually progressive, soft and non-tender mass. However, their presentation in the hand is infrequent. Some lipomas can grow considerably and their presence in the hand is associated with a variety of symptoms. Giant lipomas exhibit a size of more than 5 cm. We present a case report of a massive painless lipoma of the deep palmar space.

A 55 year old housewife presented with a one year history of an almost painless swelling over her right palm. She first noticed the swelling in the central aspect of her palm which gradually increased in size and over a period of one year, it has attained its present size. She had occasional pain while doing her daily activities and difficulty in holding any object. She had no tingling sensation or numbness over the palm or the fingers. On clinical examination, the swelling started from the wrist crease and involved both the thenar and the hypothenar region, extending up to the distal palmar crease (Figure 1). It also encroached into the first web space. On palpating the swelling, it was non-tender, soft and non-compressible with ill-defined margins and smooth surface. Patient was able to flex and extend her fingers and thumb without any restriction and there was no sensory loss. She already had a high resolution ultrasonography done for her swelling which was suggestive of lipoma. Our patient was poor and could not afford MRI scan. Hence, based on the clinical and radiological examination, we made a provisional diagnosis of lipoma and an excisional biopsy was planned. 

**Fig. 1:**
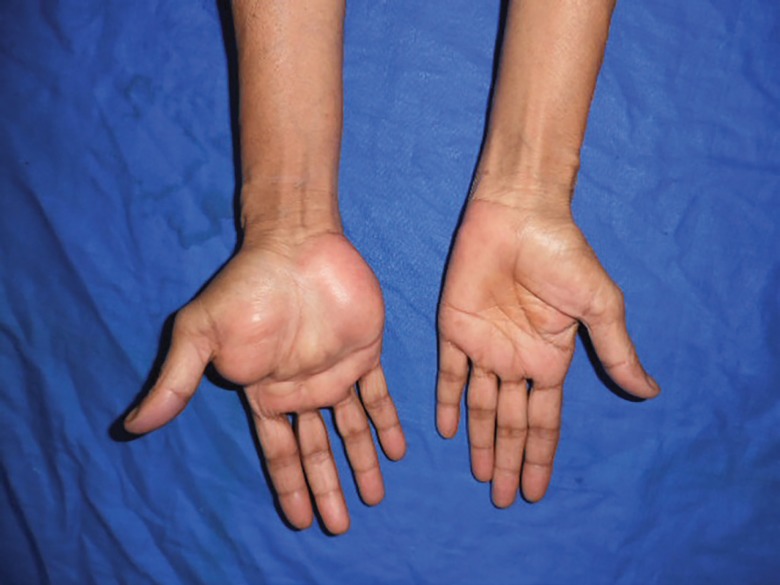
Preoperative photograph of the giant palmar lipoma (note the extent of the lesion

Operative procedure: Surgery was done under regional anesthesia. With the arm tourniquet inflated, an incision was given over the thenar crease as the swelling was most prominent in that region. Distally, this incision extended in a transverse fashion and dissection started from proximal to distal. Intra-operatively, a multi-lobulated, well encapsulated, yellowish mass was seen which was delineated on all sides. Proximally it extended into the carpal tunnel and hence it was also released. During dissection on the undersurface of the swelling, all the neurovascular structures were identified and preserved (Figure 2 and 3). In the first web space, it was separated from the adductor policies muscle. The lesion was removed en masse and sent for histo-pathological examination. Tourniquet was deflated and after achieving adequate hemostasis, skin was closed and compression dressing was applied. Post-operative course: In the postoperative period, the patient did not complaints of any restriction in the finger movements or any sensory loss. After removal of the sutures, the patient was taught passive and active mobilizing exercises. Histopathology confirmed the presence of lipoma.

**Fig. 2:**
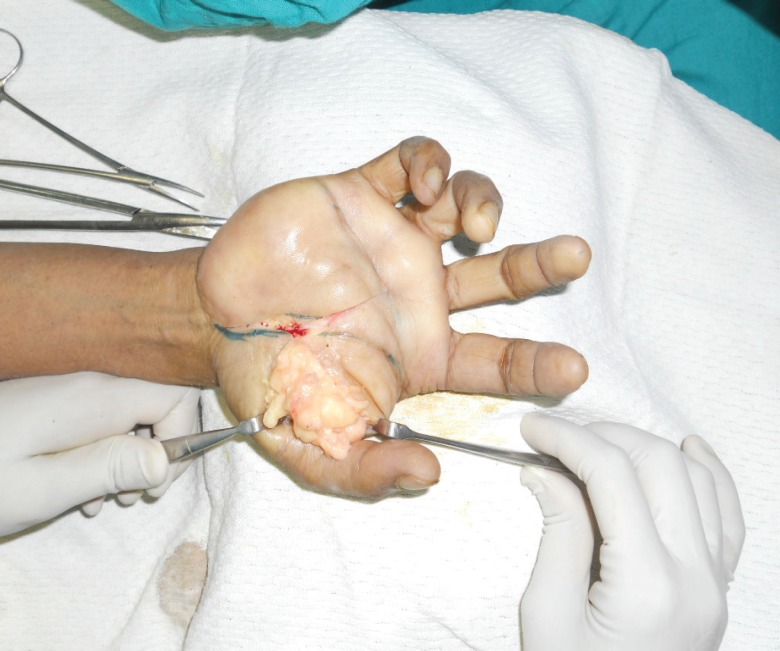
Intraoperative photograph showing the extension of lipoma in the thenar region and first web space

**Fig. 3:**
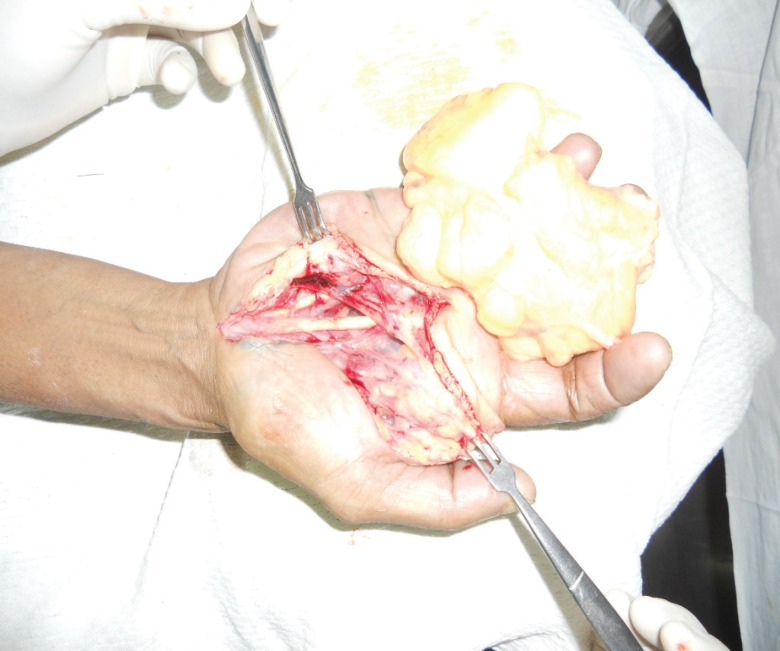
Intraoperative photograph showing the tumor mass with preserved neurovascular structures and underlying tendons.

Lipomas consist of mature fat cells, which may occur in subcutaneous, inter-muscular or intra-muscular locations.^2^ They generally progress slowly and painfully which explains their often large size at diagnosis, particularly if located deeply. Although there is a good amount of fat in the palm region, the commonest tumor of the body that arises in the fat, that is lipoma, is only rarely seen at this location.^3^ Soft tissue lipomas are categorized by anatomic location as either superficial (subcutaneous) or deep and their contour is determined by the confines of the space the tumor occupies. Fat tumors however have the ability to insinuate themselves into small recesses and thus produce tumors of any size or shape, infiltrating spaces not tightly guarded by protecting sheaths as fascia. This is especially true with tumors of the hand where lipomas occur in various anatomic locations within it. Superficial lipomas arise in the subcutaneous tissues while deep lipomas arise in the Guyon’s canal, in the carpal tunnel, and the deep palmar space.^2^

Lipoma in the hand typically presents as painless swelling and usually attains a large size by the time patient seeks medical attention. The deep-embedded and intramuscular lipomas are less defined, considerably larger in size, and much less common than their superficial counterparts due to the thick palmar fascia obscuring the true size and extent of these tumors. MRI gives the correct diagnosis in 94% of cases.^1^ Consequently, the required surgery may be more extensive than originally planned, due to the anatomy usually being distorted. Good results can be obtained with surgical treatment, but, as with large tumors located elsewhere, these require a thorough preoperative assessment.^4^ Marginal excision is usually curative and chances of recurrence are minimal. Johnson et al advised that any soft tissue tumor lump, which is greater than 5cm, should be considered as malignant until proved otherwise.^5^

But in cases of lipomas, they can be of giant size without any malignant transformation. Deep palmar lipomas are rare. Palmar lipomas usually do not cause numbness or weakness of the hand. They can be deceptively large and extensive. Simple imaging such as ultrasound is useful for a superficially located lipoma. MRI, however, provides correct diagnosis in about 94% of cases. Vital structures should be identified and preserved. With careful surgical technique the complications can be prevented. Our report emphasizes that the marginal excision of lipomas is sufficient.

## CONFLICT OF INTEREST

The authors declare no conflict of interest.
